# Antigens from the Helminth *Fasciola hepatica* Exert Antiviral Effects against SARS-CoV-2 In Vitro

**DOI:** 10.3390/ijms241411597

**Published:** 2023-07-18

**Authors:** Judit Serrat, Clara Francés-Gómez, David Becerro-Recio, Javier González-Miguel, Ron Geller, Mar Siles-Lucas

**Affiliations:** 1 Laboratory of Helminth Parasites of Zoonotic Importance (ATENEA), Institute of Natural Resources and Agrobiology of Salamanca (IRNASA-CSIC), C/Cordel de Merinas 40-52, 37008 Salamanca, Spain; judit.serrat@irnasa.csic.es (J.S.); david.becerro@irnasa.csic.es (D.B.-R.); 2 Institute for Integrative Systems Biology (I2SysBio), Universidad de Valencia-CSIC, 46980 Valencia, Spain; clarafrancesgomez@gmail.com (C.F.-G.); ron.geller@uv.es (R.G.)

**Keywords:** SARS-CoV-2, COVID-19, helminths, *Fasciola hepatica*, in vitro, antiviral

## Abstract

SARS-CoV-2, the causal agent of COVID-19, is a new coronavirus that has rapidly spread worldwide and significantly impacted human health by causing a severe acute respiratory syndrome boosted by a pulmonary hyperinflammatory response. Previous data from our lab showed that the newly excysted juveniles of the helminth parasite *Fasciola hepatica* (FhNEJ) modulate molecular routes within host cells related to vesicle-mediated transport and components of the innate immune response, which could potentially be relevant during viral infections. Therefore, the aim of the present study was to determine whether FhNEJ-derived molecules influence SARS-CoV-2 infection efficiency in Vero cells. Pre-treatment of Vero cells with a tegument-enriched antigenic extract of FhNEJ (FhNEJ-TEG) significantly reduced infection by both vesicular stomatitis virus particles pseudotyped with the SARS-CoV-2 Spike protein (VSV-S2) and live SARS-CoV-2. Pre-treatment of the virus itself with FhNEJ-TEG prior to infection also resulted in reduced infection efficiency similar to that obtained by remdesivir pre-treatment. Remarkably, treatment of Vero cells with FhNEJ-TEG after VSV-S2 entry also resulted in reduced infection efficiency, suggesting that FhNEJ-TEG may also affect post-entry steps of the VSV replication cycle. Altogether, our results could potentially encourage the production of FhNEJ-derived molecules in a safe, synthetic format for their application as therapeutic agents against SARS-CoV-2 and other related respiratory viruses.

## 1. Introduction

Severe acute respiratory syndrome coronavirus 2 (SARS-CoV-2), the etiological agent of the coronavirus disease 2019 (COVID-19) pandemic, has thus far affected over 600 million people worldwide, caused almost seven million deaths [1] and inflicted unprecedented damage to the global economy [2]. SARS-CoV-2 is an enveloped, single-stranded RNA virus of the *Coronaviridae* family that preferentially infects bronchial epithelial cells, pneumocytes and cells of the upper respiratory tract [3]. Cellular entry of SARS-CoV-2 is mediated by the envelope glycoprotein spike (S), which binds to its target cell receptor, the surface-exposed angiotensin-converting enzyme 2 (ACE2), promoting the release of the virion’s genome into the host cell cytoplasm [4]. SARS-CoV-2 cell entry preferentially occurs via clathrin-mediated endocytosis [5], although direct fusion at the plasma membrane can also occur [3]. Innate immune responses lead by type I interferons (IFNs) are induced early after infection and correlate with a reduction in SARS-CoV-2 viremia [6]. Severe cases of COVID-19 are characterized by excessive innate and dysregulated adaptive immune responses against SARS-CoV-2 that result in a hyper-inflammatory state characterized by the over-expression of pro-inflammatory cytokines [7].

Helminth parasites are amongst the widest spread human infections, affecting over two billion people worldwide [8]. Therefore, a substantial overlap exists between them and other infectious agents and it is consequently highly expected that helminth infections have an impact on host responses to concurrent infectious diseases. Our group and others have postulated that immune modulation by helminth parasites may influence the hyper-inflammatory response that underlies COVID-19 severity and lethality [9,10,11], which has recently been supported by data showing that CD4+ T cell-mediated responses to SARS-CoV-2 are alleviated by helminth antigens [12]. In addition to modulating host immune responses to viruses, some helminths can also directly influence viral infection efficiency (rev. in [13,14,15]). This encourages further research considering that, despite a large number of SARS-CoV-2 inhibitors being explored, the identification of specific antiviral treatments for COVID-19 remains an urgent need [16]. In this respect, a wide variety of compounds that potentially inhibit SARS-CoV-2 enzymes or virus entry, including monoclonal antibodies, interferons and repurposed drugs, have been developed as antiviral therapies for COVID-19 with a rather disappointing outcome [17].

*Fasciola hepatica* is a helminth parasite with a complex life cycle that includes an invertebrate intermediate host (water snails), where the asexual forms of the parasite develop and are shed, and a vertebrate definitive host, typically ruminants raised as livestock. Because *F. hepatica* can also complete its lifecycle in humans, human fasciolosis is considered a major food-borne zoonosis of public health concern [8,18]. The definitive host becomes infected by ingesting metacercariae in water or attached to water plants, which excyst in the duodenum and release the newly excysted juveniles (FhNEJs). Within the next two to three hours after excystment, FhNEJs cross the intestinal wall. Then, the parasites crawl up the peritoneum and develop into immature flukes that burrow through hepatic tissue to finally reach the biliary ducts, where adult worms develop and produce eggs that are shed with feces [19].

Based on previous data from our lab showing that FhNEJs induce proteomic changes in host cells related to vesicle-mediated trafficking and an upregulation of proteins of the type I IFN pathway [20,21], we recently highlighted the potential influence of *F. hepatica* on SARS-CoV-2 infection success [10]. Given that endocytosis and type I IFN-mediated innate immune responses are directly involved in SARS-CoV-2 cell entry and/or replication [5,6], FhNEJs may modulate pathways in host cells that are important during SARS-CoV-2 infection. In order to test this hypothesis, we evaluated the antiviral effects of FhNEJ protein extracts and specific recombinant proteins. To this end, we used an in vitro model for SARS-CoV-2 entry based on vesicular stomatitis viral particles pseudotyped with the S protein (VSV-S2) and validated these results in an in vitro model using genuine SARS-CoV-2 infections.

## 2. Results

### 2.1. FhNEJ-TEG Reduces Infection Efficiency of VSV-S2 in Vero Cells In Vitro

**Figure 1:**
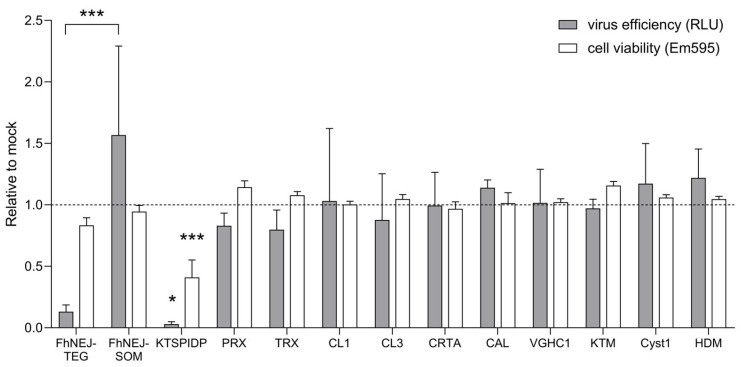
Compound screen to test the ability of *F. hepatica*-derived molecules to regulate virus entry into Vero cells using VSV-S2 pseudotyped viral particles. Vero cells were pre-treated with the indicated compounds (20 µg/mL) for two hours prior to addition of VSV-S2 viral particles. Twenty-four hours after infection, virus entry was addressed by measuring luminescence derived from luciferase expression (RLU, relative light units; grey bars). Cell viability was addressed by measuring resazurin conversion to resofurin (Em595) by live cells (white bars). Bars indicate the mean of three technical replicates ± SD calculated relative to control (mock)-treated cells, and asterisks indicate significant differences between each group and control (mock)-treated cells (* *p* ≤ 0.05, *** *p* ≤ 0.001; one-way ANOVA). FhNEJ-TEG, tegument-enriched antigenic extract of FhNEJs; FhNEJ-SOM, somatic-enriched antigenic extract of FhNEJs; KTSPIDP, kazal-type serine protease inhibitor domain protein; PRX, peroxiredoxin; TRX, thioredoxin; CL1, cathepsin L1; CL3, cathepsin L3; CRTA, cholecystokinin receptor type A; CAL, catenin alpha-like protein; VGHC1, voltage-gated hydrogen channel 1; KTM, kunitz-type molecule; Cyst1, cystatin 1; HDM, helminth defense molecule.

Eleven recombinant proteins that are biologically relevant for *F. hepatica*, consisting of the kazal-type serine protease inhibitor domain protein (KTSPIDP), peroxiredoxin (PRX), thioredoxin (TRX), cathepsins L1 (CL1) and L3 (CL3), cholecystokinin receptor type A (CRTA), catenin alpha-like protein (CAL), voltage-gated hydrogen channel 1 (VGHC1), kunitz-type molecule (KTM), cystatin 1 (Cyst1) and helminth defense molecule (HDM), were included in this study, together with tegument (FhNEJ-TEG)- and somatic (FhNEJ-SOM)-enriched antigenic extracts of FhNEJs, which preferentially contain proteins of the parasite surface or internal organs, respectively. Treatment of Vero cells before VSV-S2 infection with FhNEJ-TEG and KTSPIDP caused a clear reduction, although not statistically significant, in cell entry of VSV-S2, as measured by luciferase activity (Figure 1). Because the incubation of cells with KTSPIDP resulted in statistically significant cell toxicity compared to their untreated counterparts (Figure 1), this protein was discarded for subsequent analyses. Similar results were obtained by infecting a human airway epithelial cell line (Calu-3) with VSV-S2, although FhNEJ-SOM did cause a reduction in infection efficiency in this case (Figure S1).

**Figure 2:**
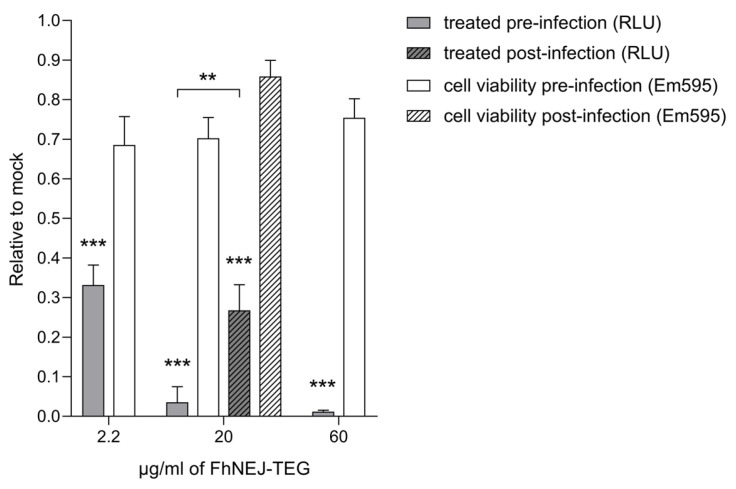
FhNEJ-TEG inhibits VSV-S2 entry into Vero cells in a dose-dependent manner and post-entry steps of the VSV replication cycle. Vero cells were treated with increasing concentrations of FhNEJ-TEG (2.2 µg/mL to 60 µg/mL) for two hours prior to addition of VSV-S2 pseudotyped viral particles (solid bars). Alternatively, cells were infected with VSV-S2 and FhNEJ-TEG (20 µg/mL) was added one hour after infection and incubated for two hours (hatched bars). Infection efficiency was measured by luciferase activity (RLU, relative light units; grey bars). Cell viability was addressed by measuring resazurin conversion to resofurin (Em 595; white bars) by live cells. Bars indicate the mean of three technical replicates ± SD calculated relative to control (mock)-treated cells, and asterisks indicate significant differences between each condition and control (mock)-treated cells (** *p* ≤ 0.01, *** *p* ≤ 0.001; one-way ANOVA). FhNEJ-TEG, tegument-enriched antigenic extract of FhNEJs.

FhNEJ-TEG was then assayed at different concentrations using the same experimental setting, showing that its antiviral effect is dose-dependent without causing a proportional increase in cell toxicity (Figure 2). When FhNEJ-TEG was added to Vero cells after VSV-S2 infection, the abovementioned antiviral effects were also observed, although they were significantly lower than those achieved by treating cells before virus addition (Figure 2).

### 2.2. FhNEJ-TEG Inhibits SARS-CoV-2 Infection of Vero Cells In Vitro

**Figure 3:**
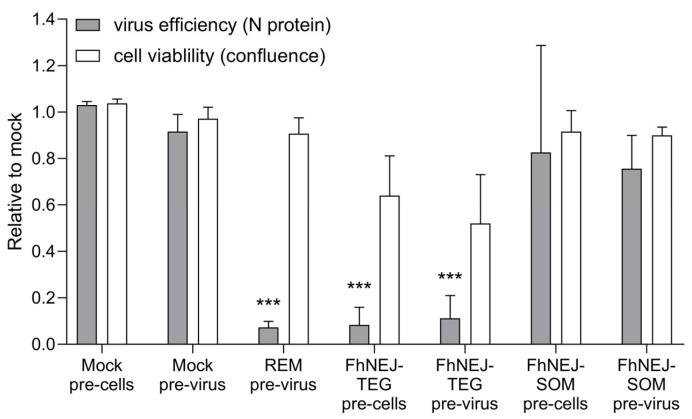
FhNEJ-TEG reduces infection efficiency by live SARS-CoV-2 in Vero cells in vitro. Vero cells were treated with FhNEJ-TEG (50 μg/mL) or FhNEJ-SOM (50 μg/mL) for two hours prior to addition of SARS-CoV-2 at a multiplicity of infection (MOI) of 0.1 (‘pre-cells’). Alternatively, an aliquot of SARS-CoV-2 containing an MOI of 0.1 was incubated with the indicated compounds for one hour prior to addition to Vero cells (‘pre-virus’). In both settings, cells were processed for immunofluorescence analysis on a live-cell microscope 24 h after virus addition. Infection efficiency was estimated by SARS-CoV-2 N protein expression and cell viability was assessed by cell confluency. Relative fluorescence and cell viability were calculated relative to control (mock)-treated cells (grey bars). Treatment of viral particles with remdesivir (20 µM) for one hour prior to addition to target cells served as a positive control for antiviral activity. Bars represent the mean of three biological replicates ± SEM, every replicate representing the average of three technical replicates. Asterisks indicate significant differences between each group and their mock-treated counterpart cells (*** *p* ≤ 0.001; one-way ANOVA). DMEM, Dulbecco’s Modified Eagle Medium; REM, remdesivir; FhNEJ-TEG, tegument-enriched antigenic extract of FhNEJs; FhNEJ-SOM, somatic-enriched antigenic extract of FhNEJs.

In this assay, we pre-incubated either the virus or the cells with FhNEJ-TEG or FhNEJ-SOM in order to check for the effects that these compounds have both in the cells and the virus prior to infection. Our results show that FhNEJ-TEG pre-treatment of Vero cells significantly inhibits SARS-CoV-2 infection without causing substantial changes in cell viability (Figure 3). In addition, FhNEJ-TEG antiviral effects against SARS-CoV-2 are also significant when the virus is incubated with this extract prior to cell infection (Figure 3). In both settings, the observed antiviral effects are similar to those elicited by virus pre-treatment with remdesivir, which serves as a positive control for antiviral activity. Finally, and in line with our results with VSV-S2 viral particles (Figure 1), FhNEJ-SOM failed to inhibit SARS-CoV-2 infection of Vero cells (Figure 3).

## 3. Discussion

Coinfections are increasingly recognized as important drivers of disease dynamics [22]. However, little is known about host factors that are decisive for COVID-19 incidence and severity, especially concerning co-occurrence of SARS-CoV-2 with other infectious agents. In this regard, it has been hypothesized that helminth coinfections may have an impact on SARS-CoV-2 outcomes [9,10,11]. Nevertheless, and despite the large body of knowledge supporting an influence of helminths on the outcomes of concomitant viral infections (rev. in [13,14,15]), the role of these organisms and their derived compounds on SARS-CoV-2 infection has not been reported yet. The present study aims at filling this knowledge gap by investigating whether the helminth parasite *F. hepatica* expresses molecules that are capable of modulating SARS-CoV-2 infection efficiency.

During *F. hepatica* infection, a transient cellular immune response driven by IFN-γ in the very early steps of infection has been described [23], which could trigger a Th1 response with potential antiviral effects [24]. In addition, an early sensing of invading helminths in epithelial tissues causes the release of alarmins such as interleukin-33 [25], which elicits antiviral CD8+ T cell-dependent immune responses [26]. In line with this, previous studies by us showed that the in vitro interaction between live FhNEJs and primary epithelial intestinal cells resulted in the upregulation of molecules related to type I IFN-related innate responses [21]. Importantly, an increase in type I IFN signaling in the lung has been proposed to be protective against respiratory syncytial virus in mice coinfected with the helminth parasite *Heligmosomoides polygyrus* [27]. In addition to IFN-related pathways, we also observed that FhNEJs induce a downregulation of proteins related to vesicle-mediated trafficking in epithelial cells [20,21], which could be relevant in the context of the present study considering that the main entry route of SARS-CoV-2 in Vero cells is endocytosis [28]. Based on this, we decided to use FhNEJs as the source of potential antiviral compounds.

In the first screening, we used an in vitro system based on the infection of Vero cells with SARS-CoV-2 pseudotyped viral particles (VSV-S2), which bypass the need for high-containment biological facilities and have been successfully used in drug repurposing screens for SARS-CoV-2 [16,29]. Because no information is available about specific molecules of helminth parasites that could interfere with viral infections, we decided to use FhNEJ recombinant proteins that are highly expressed in this developmental stage and/or have been described as immunomodulatory molecules. These included PRX, TRX, HDM, CL1 and CL3, major components of the FhNEJ secretome that play an important role in the modulation of innate immune responses [30,31,32]; KTM, Cys1 and KTSPIDP, which are cathepsin protease inhibitors [33,34,35] that can be relevant during viral infection processes [36]; and VGCH1, CRTA and CAL, which are tegument proteins important for FhNEJ biology and potentially involved in host–parasite interactions [20]. Additionally, and considering that the abovementioned results originated from the stimulation of cells with live FhNEJs and not with specific molecules, we decided to use protein extracts of two antigenic compartments of FhNEJs: FhNEJ-SOM, which contains proteins derived from internal organs, and FhNEJ-TEG, which is enriched for surface proteins of the parasites. It is worth mentioning that the tegument of *F. hepatica*, like that of other parasitic trematodes, is a biologically active and metabolically complex matrix that represents a site where considerable biochemical, physiological and immune interplay takes place between the fluke and its host [32]. Regarding the specific composition of these extracts, some studies have addressed divergent sets of molecules by using different -omic approaches (rev. in [37])

A tendency towards a reduction in the infection efficiency of VSV-S2 viral particles in Vero cells was observed only when cells were pre-incubated with FhNEJ-TEG, and similar results were obtained when cells were treated with FhNEJ-TEG after VSV-S2 addition. These findings indicate that FhNEJ-TEG could act hampering both Spike-mediated virus entry and post-entry processes related to VSV replication. Because post-entry steps of the VSV-S2 cycle are specific for VSV [4] and our objective was to investigate the potential of FhNEJ molecules to specifically block SARS-CoV-2 cell entry, we considered that the observed post-entry antiviral effects were beyond the scope of the present study and were thus not further investigated.

The results obtained using VSV-S2 pseudotyped viral particles were validated using genuine SARS-CoV-2 infection of Vero cells. This experiment showed that FhNEJ-TEG treatment of either the cells or the virus prior to infection leads to a statistically significant and strong decrease in SARS-CoV-2 infection efficiency. Conversely, and consistent with the results obtained using VSV-S2, FhNEJ-SOM did not show any detectable antiviral effects. It is noteworthy that the antiviral activity of FhNEJ-TEG in this model was similar to that obtained upon remdesivir treatment, one of the very few drugs that are currently approved to treat COVID-19 patients [38,39].

Mechanistically, we envision that an inhibition of endocytosis-mediated virus entry and/or the stimulation of paracrine, IFN-related innate responses involved in viral replication and clearance by FhNEJ-TEG proteins might underlie the observed antiviral effects in Vero cells. This would be supported by previous studies by us showing that the interaction between live FhNEJs and primary cells of the small intestine leads to a differential modulation of signaling routes related to endocytosis and type I IFN signaling [20,21]. Because Vero cells are deficient in type I IFN signaling due to genetic mutations [40,41,42], the most plausible scenario is that FhNEJ-TEG proteins might modulate endocytosis-related pathways in these cells that are needed for Spike-mediated virus entry. Indeed, studies by others have shown that the main entry route of SARS-CoV-2 in Vero cells is endocytosis [28]. Alternatively, additional innate antiviral mechanisms not strictly related to type I IFN signaling could be playing a role in VSV-S2 and SARS-CoV-2 restriction in Vero cells, such as the expression of type III IFNs (IFN-λ1-4). These antiviral mediators are encoded by genes located at a different chromosome than their type I counterparts [43] and they are produced by Vero cells upon viral infection [44]. Interestingly, tissue-specific expression of type III IFNs restricts their activity to mucosal epithelial barriers [45,46], which makes them particularly relevant in the context of respiratory viral infections, including that by SARS-CoV-2 [47]. Notwithstanding these points, we observed decreased VSV-S2 infection efficiency upon FhNEJ-TEG pre-treatment in Calu-3 cells. As opposed to Vero cells, Calu-3 cells express type I IFNs in response to viral infections [48], supporting our initial hypothesis that the stimulation of type I IFN mediators may also participate in the potential antiviral effects of FhNEJs.

In striking contrast with the results obtained in Vero cells, FhNEJ-SOM did cause a reduction in VSV-S2 entry into Calu-3 cells. The fact that VSV-S2 entry depends on different pathways in Vero and Calu-3 cells due to differing expression of Spike-activating proteases harmonizes these apparently conflicting results. The Spike glycoprotein needs to be cleaved after ACE2 receptor engagement by specific host proteases (the transmembrane protease serine 2 (TMPRSS2) at the cell surface or cathepsin L at the endosomal compartment) to activate its fusogenic potential [49]. Consequently, the cell-specific expression pattern of these proteases dictates the entry route used by SARS-CoV-2 in different cell types. Given that Calu-3 cells express high levels of TMPRSS2 and low levels of active cathepsin L, whereas the opposite is true for Vero cells, the main entry route of SARS-CoV-2 in these cells is the plasma membrane and endocytosis, respectively [28]. These points reinforce the notion that FhNEJ-TEG could be influencing cellular pathways related to endocytosis, resulting in reduced Spike-mediated virus entry. The fact that FhNEJ-TEG also interferes with VSV-S2 entry into Calu-3 cells does not contradict this idea because these cells partially depend on the endosomal pathway for SARS-CoV-2 cell entry despite prominent TMPRSS2 expression [28]. The possibility that FhNEJ-TEG may contain molecules that modulate endocytosis is compelling given that drugs that target the endocytic pathway have been proposed as promising agents to treat SARS-CoV-2 [50]. That said, further research needs to be conducted to unravel the precise molecular mechanisms that underlie the observed antiviral effects.

Finally, none of the recombinant proteins tested in this system showed antiviral effects. Whether this is due to differences in post-translational modifications and 3D structure of the recombinant proteins compared with their native counterparts or to the need of interactions among several FhNEJ molecules to exert an antiviral effect remains an open question.

## 4. Materials and Methods

### 4.1. F. hepatica Compounds

FhNEJs were obtained by in vitro stimulation of the excystment of five thousand *F. hepatica* metacercariae (Ridgeway Research Ltd., St Briavels, UK) as previously described [51]. After two washes in phosphate saline buffer (PBS), FhNEJ-TEG and FhNEJ-SOM were obtained as described elsewhere [20,21] and frozen at −80 °C until use. In brief, FhNEJ-TEG and FhNEJ-SOM proteins were extracted with 1% Nonidet P40 substitute (NP40; Sigma, St. Louis, MO, USA) and RIPA buffer (Sigma, St. Louis, MO, USA), respectively. Prior to cell stimulation, detergents were removed from FhNEJ-TEG and FhNEJ-SOM as follows: NP40 was removed using SM-2 Adsorbent Bio-Beads (BioRad, Hercules, CA, USA) following the manufacturer’s protocol and FhNEJ-SOM proteins were purified using the ReadyPrep 2-D Cleanup kit (BioRad, Hercules, CA, USA) and resuspended in PBS. Protein concentrations were determined using the Pierce BCA Protein Assay kit (Thermo Fisher Scientific, Waltham, MA, USA). Additionally, eleven recombinant proteins of *F. hepatica*, involved in host–parasite relationships and considered important for parasite development, were used in this study. Recombinant versions of *F. hepatica* PRX, TRX, CL1, CL3, KTM, HDM and Cyst1 were kindly given by John P. Dalton’s laboratory at the University of Galway and solubilized in either PBS (2.5 mM NaH_2_PO_4_, 7.5 mM Na_2_HPO_4_, 145 mM NaCl) or physiological saline (0.9% NaCl). Additionally, total or partial protein sequences of FhNEJ proteins were subcloned and obtained in a soluble format for VGHC1, CRTA, CAL and KTSPIDP. Briefly, in silico analysis of the VGHC1 sequence BN1106_s2883B000034.mRNA-1 revealed a transmembrane structure, so the extracellular fragment was made recombinant to ensure protein solubility. The same applied to CRTA (BN1106_s923B000146.mRNA-1), thus a small fragment of this protein comprising amino acids 180–234 was produced and tagged with thioredoxin and His. Due to its large size, only a His-tagged carboxy-terminus domain of CAL (BN1106_s45B000418.mRNA-1) comprising amino acids 491–901 was produced; and given that the KTSPIDP sequence (BN1106_s800B000486.mRNA-1) revealed no transmembrane domains, the whole protein excluding the last 50 amino acids was obtained and tagged with His. All the recombinant proteins were produced in *E. coli* by the company GenScript Biotech (Rijswijk, The Netherlands) and solubilized in either PBS (2.5 mM NaH_2_PO_4_, 7.5 mM Na_2_HPO_4_, 145 mM NaCl)- or Tris (50 mM Tris-HCl, 150 mM NaCl)-based buffers (pH 7.4–8.0) containing 10% glycerol, except for the KTSPIDP buffer (Tris-based), which also included 0.5 M L-Arg to increase protein solubility.

### 4.2. Pseudotyped VSV-S2 Infection Assay

Human embryonic kidney cells (HEK293, CRL-1573; American Type Culture Collection, Manassas, Virginia), African green monkey kidney cells (Vero, CCL-81; American Type Culture Collection, Manassas, VA, USA) and human airway epithelial cells (Calu-3, HTB-55; American Type Culture Collection, Manassas, Virginia) were maintained in Dulbecco’s Modified Eagle Medium (DMEM; Thermo Fisher Scientific, Waltham, MA, USA) supplemented with 10% fetal bovine serum (FBS; Thermo Fisher Scientific, Waltham, MA, USA) and 1% penicillin-streptomycin (Thermo Fisher Scientific, Waltham, MA, USA) in a 5% CO_2_ humidified atmosphere at 37 °C. Pseudotyped VSV carrying a codon-optimized spike protein of the Wuhan SARS-CoV-2 strain (VSV-S2) encoding both green fluorescent protein and firefly luciferase was produced in HEK293 cells and tittered on Vero cells as previously described [52,53]. For antiviral assays, 10^4^ target cells per well were seeded the day before on 96-well plates and *F. hepatica* compounds were added at different concentrations for 2 h at 37 °C in a volume of 100 µL or left untreated, followed by addition of a small aliquot containing 1000 focus-forming units (ffu) of VSV-S2 viral particles. Sixteen hours after VSV-S2 addition, cell viability was assessed by the addition of resazurin (Sigma, St. Louis, MO, USA) to a final concentration of 0.4 mg/mL, incubation for 1 h at 37 °C and fluorescence reading on a microplate reader (Tecan, Männedorf, Switzerland) with excitation and emission filters of 535 nm and 595 nm, respectively. After this, infection efficiency was quantified using the Pierce Firefly Luc One-Step Glow Assay Kit (Thermo Fisher Scientific, Waltham, MA, USA) and luminescence was read on a microplate reader (Tecan, Männedorf Switzerland). To assess the influence of FhNEJ-TEG on viral replication efficiency after virus entry, cells were infected with VSV-S2 for 1 h, after which 20 µg/mL of FhNEJ-TEG was added. Virus infection and toxicity were quantified as indicated above. All the experiments were performed in technical triplicates and antiviral activity and cell viability were calculated relative to control (mock)-treated cells.

### 4.3. SARS-CoV-2 Infection Assay

On day 0, 10^4^ Vero cells/well were seeded in tissue-culture-treated 96-well plates in DMEM (Thermo Fisher Scientific, Waltham, MA, USA) containing 10% FBS (Thermo Fisher Scientific, Waltham, MA, USA). On day 1, either the virus or the cells were pretreated with the compounds as follows: cell pre-treatment was performed by adding the desired compound diluted in DMEM (Thermo Fisher Scientific, Waltham, MA, USA) with 2% FBS (Thermo Fisher Scientific, Waltham, MA, USA) to the cells for 2 h at 37 °C, after which a small aliquot of the SARS-CoV-2 virus at a multiplicity of infection (MOI) of 0.1 was added and incubated for 24 h at 37 °C. Alternatively, virus pre-treatment was performed by mixing an aliquot of SARS-CoV-2 (MOI of 0.1) with the desired compound diluted in DMEM (Thermo Fisher Scientific, Waltham, MA, USA) with 2% FBS (Thermo Fisher Scientific, Waltham, MA, USA) for 1 h at 37 °C, after which the virus in the compound mix was added to the cells and incubated for 24 h at 37 °C. FhNEJ-TEG and FhNEJ-SOM were used at 50 µg/mL. Remdesivir, an adenosine analogue that showed antiviral activity in vitro against SARS-CoV-2 [54], was used at 20 µM as a positive control for antiviral activity. Mock-treated cells were used as negative controls. On day 2, virus entry was assessed by immunofluorescence of SARS-CoV-2 nucleocapsid (N) protein, a structural protein encoded by the SARS-CoV-2 genome [55]. Briefly, growth medium was removed from the wells and cells were fixed by adding 100 µL of 100% methanol for 30 min at room temperature. After two washes with PBS, 50 µL of incubation buffer consisting of 3% FBS (Thermo Fisher Scientific, Waltham, MA, USA) and 0.3% Triton-X100 (Sigma, St. Louis, MO, USA) in PBS were added to each well and incubated for 1 h at room temperature. N protein expression was detected by adding 50 µL of anti-N SARS-CoV-2 primary antibody (Genetex, Irvine, CA, USA) diluted 1:2000 in incubation buffer followed by two washes with PBS and the addition of 50 µL/well of mouse anti-rabbit IgG-CFL 488 (Santa Cruz Biotechnology, Dallas, TX, USA) diluted 1:1000 in incubation buffer. Antibody incubations were conducted at room temperature for 1 h. After two washes in PBS, virus infection and cell viability were quantified on a live-cell microscope (Incucyte S3 Live-Cell Analysis Instrument; Sartorius, Göttingen, Germany) by analyzing green fluorescence intensity and cell confluency, respectively. Green fluorescence intensity was measured within the cell cytoplasm by using frames of equal areas in every well (one frame per well spanning almost the entire well area and excluding the round plate edges). The relative cell confluency or the relative antiviral effect were obtained by dividing either the confluency data or the integrated fluorescence in the green channel by those obtained from infected, mock-treated cells (see Figure S2 for examples of images). All experiments were performed in triplicate, with each experiment comprising three technical replicates. The SARS-CoV-2 isolate was kindly given by Sonia Zúñiga, Isabel Sola and Luis Enjuanes from the Spanish National Centre for Biotechnology (CNB-CSIC) and it contained the Wuhan-Hu-1 strain (GenBank MN908947) with a silent mutation C3037 > T and two mutations leading to amino acid changes C14408 > T (in the nsp12 RNA-dependent RNA polymerase) and A23403 > G (D614G in the S protein). Infections with live SARS-CoV-2 were carried out at the Biosafety Level 3 Facility of the Fundación para el Fomento de la Investigación Sanitaria y Biomédica (FISABIO) in Valencia, Spain. All experiments were performed in biological triplicates, every replicate representing the average of three technical replicates, and data are shown relative to control (mock)-treated cells.

### 4.4. Statistical Analysis

Statistical analyses were performed with the R Commander package [56]. Comparison between three or more groups used an Analysis of Variance (ANOVA) test followed by Tukey’s post hoc analysis for pair-wise comparisons. Asterisks indicate significant differences between the corresponding experimental groups (* *p* ≤ 0.05, ** *p* ≤ 0.01, *** *p* ≤ 0.0001). Unless otherwise stated, the differences are not significant.

## 5. Conclusions

In conclusion, our results show that the tegument-enriched antigenic fraction of FhNEJs contains proteins that are capable of altering the infectivity of pseudotyped and live SARS-CoV-2 viral particles in Vero cells. These results emphasize the potential of FhNEJ molecules as therapeutic agents against SARS-CoV-2 and other emerging respiratory viruses.

## Data Availability

Not applicable.

## Supplementary Materials

The following supporting information can be downloaded at https://www.mdpi.com/article/10.3390/ijms241411597/s1.
