# Supplementary material for: Antigens from the Helminth Fasciola hepatica Exert Antiviral Effects against SARS-CoV-2 In Vitro
Source: Int J Mol Sci. 2023 Jul 18;24(14):11597. doi: 10.3390/ijms241411597 (PMC10380311; doi:10.3390/ijms241411597)
Supplement: Supplementary file 1 [file ijms-24-11597-s001.zip › Figure S1.pdf]

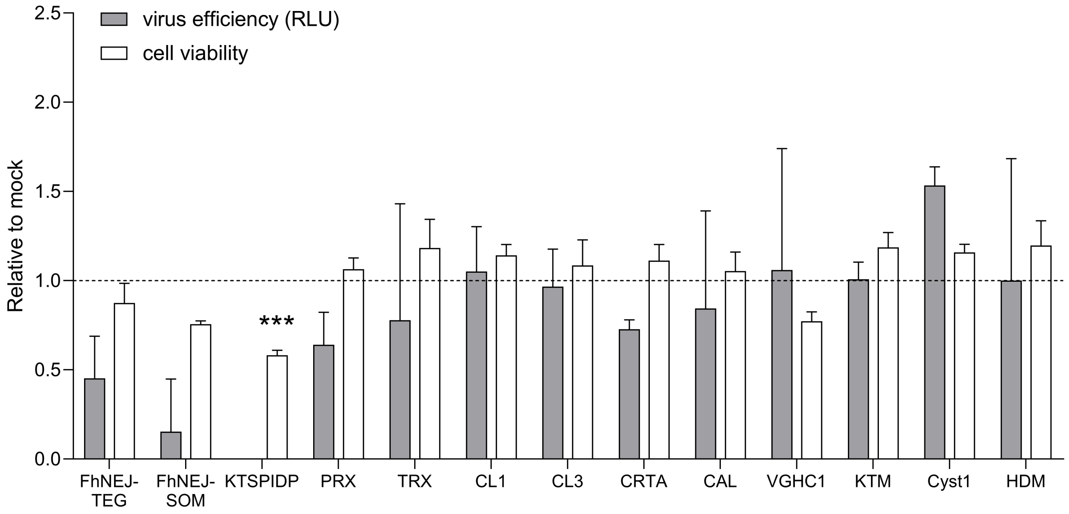

Figure S1. Related to Figure 1. Compound screen to test the ability of *Fasciola hepatica*-derived molecules to regulate virus entry into Calu-3 cells using VSV-S2 pseudotyped viral particles. Calu-3 cells were pre-treated with the indicated compounds (20 µg/ml) for two hours prior to addition of VSV-S2 viral particles. Twenty-four hours after infection, virus entry was addressed by measuring luminescence derived from luciferase expression (RLU, relative light units; grey bars). Cell viability was addressed by measuring resazurin conversion to resofurin by live cells (white bars). Bars indicate the mean of three technical replicates ± SD calculated relative to control (mock)-treated cells, and asterisks indicate significant differences between every group and control (mock)-treated cells (***$p \leq 0.001$; one-way ANOVA). FhNEJ-TEG, tegument-enriched antigenic extract of FhNEJs; FhNEJ-SOM, somatic-enriched antigenic extract of FhNEJs; KTSPIDP, kazal-type serine protease inhibitor domain protein; PRX, peroxiredoxin; TRX, thioredoxin; CL1, cathepsin L1; CL3, cathepsin L3; CRTA, cholecystokinin receptor type A; CAL, catenin alpha-like protein; VGHC1, voltage-gated hydrogen channel 1; KTM, kunitz-type molecule; Cyst1, cystatin 1; HDM, helminth defense molecule.
