# Supplementary material for: Antigens from the Helminth Fasciola hepatica Exert Antiviral Effects against SARS-CoV-2 In Vitro
Source: Int J Mol Sci. 2023 Jul 18;24(14):11597. doi: 10.3390/ijms241411597 (PMC10380311; doi:10.3390/ijms241411597)
Supplement: Supplementary file 1 [file ijms-24-11597-s001.zip › Figure S2.pdf]

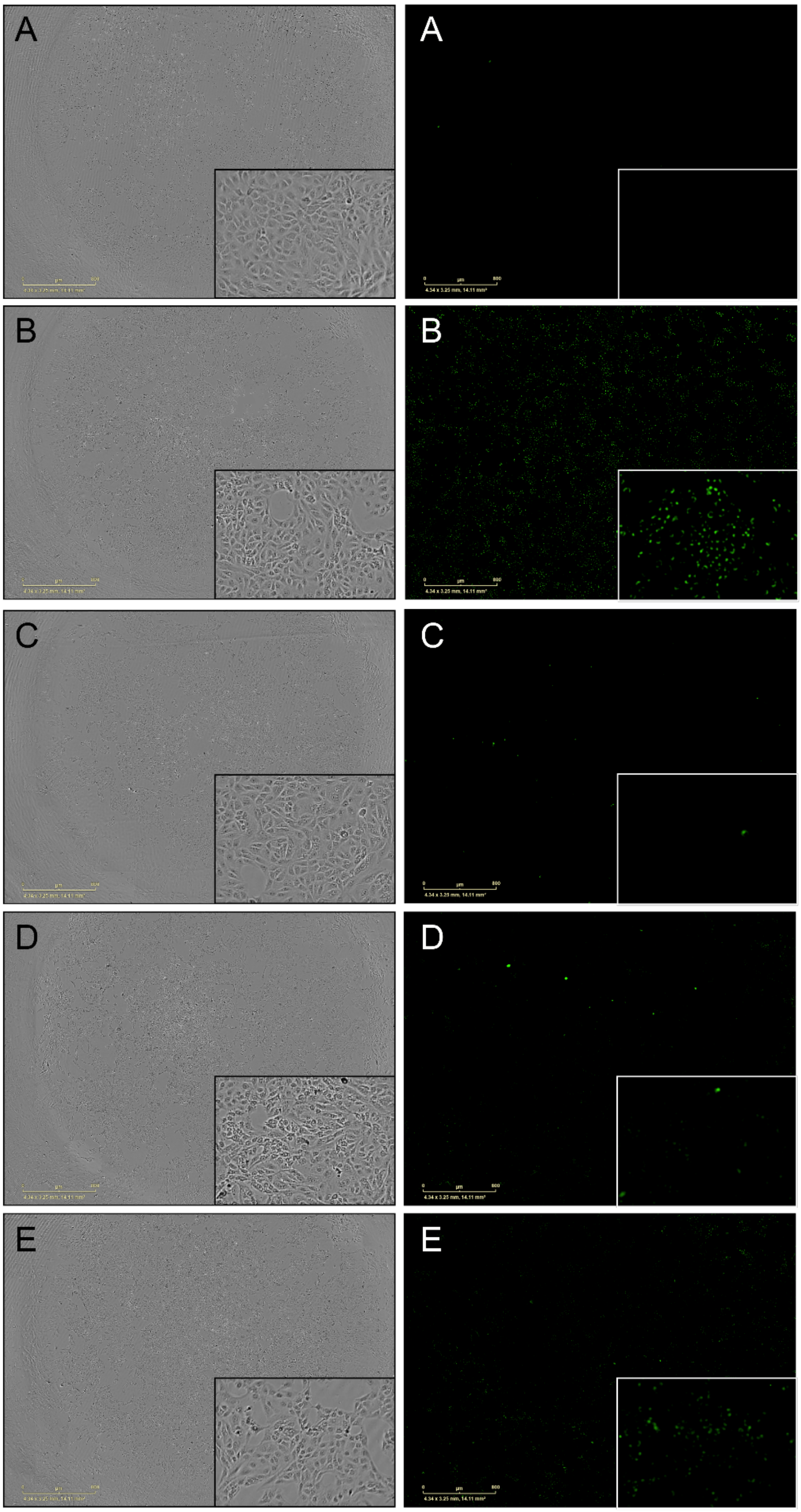

Figure S2. Related to Figure 3. Quantification of live SARS-CoV-2 infection efficiency. Example pictures of Vero cells left uninfected (A), mock-treated (B) prior to infection with SARS-CoV-2, infected with virus pre-treated with remdesivir (C) and pre-treated with FhNEJ-TEG (D) or FhNEJ-SOM (E) prior to infection with SARS-CoV-2. Left panels show the bright field images, right panels show the green fluorescence channel (CFL 488) and small panels show an example field with increased magnification for visualization purposes.
